# Multi-Force Bio-Active™ Archwires and Various Contemporary NiTi Multi-Force Archwires: Properties and Characteristics—A Review

**DOI:** 10.3390/ma17112603

**Published:** 2024-05-28

**Authors:** Angelina Stoyanova-Ivanova, Valeri Petrov, Jorge N. R. Martins, Laura Andreeva, Velizar Georgiev

**Affiliations:** 1 G. Nadjakov Institute of Solid State Physics, Bulgarian Academy of Sciences, 72 Tzarigradsko Chaussee, 1784 Sofia, Bulgaria; angelina@issp.bas.bg (A.S.-I.); velizar@issp.bas.bg (V.G.); 2 Faculty of Dental Medicine, Medical University of Sofia, St. G. Sofiiski Blvd., 1431 Sofia, Bulgaria; v.petrov@fdm.mu-sofia.bg (V.P.); laura_andreeva@fdm.mu-sofia.bg (L.A.); 3 Faculdade de Medicina Dentária, Universidade de Lisboa, 1600-277 Lisboa, Portugal; 4 LIBPhys-FCT UID/FIS/04559/2013, 1600-277 Lisboa, Portugal; 5 Grupo de Investigação em Bioquimica e Biologia Oral (GIBBO), Unidade de Investigação em Ciências Orais e Biomédicas (UICOB), 1600-277 Lisboa, Portugal; 6 Centro de Estudos de Medicina Dentária Baseada na Evidência (CEMDBE), 1600-277 Lisboa, Portugal

**Keywords:** bio-active archwires, materials, mechanical properties, multi-force archwires, thermal properties

## Abstract

The manufacturing of orthodontic archwires made from NiTi alloy has undergone numerous changes from the second half of the last century to modern times. Initially, superelastic-active austenitic NiTi alloys were predominant, followed by thermodynamic-active martensitic NiTi alloys, and, finally, the most recent development was graded thermodynamic alloys. These advancements have been the subject of extensive investigation in numerous studies, as they necessitated a deeper understanding of their properties. Furthermore, it is imperative that we validate the information provided by manufacturers regarding these archwires through independent studies. This review evaluates existing studies on the subject with a specific focus on the Bio-active multi-force NiTi archwire, by examining its mechanical, thermal, and physicochemical properties before and after clinical use. This archwire consists primarily of Ni and Ti, with traces of Fe and Cr, which release graduated, biologically tolerable forces which increase in a front-to-back direction and are affected by the temperature of the environment they are in. The review provides information to practicing orthodontists, facilitating informed decisions regarding the selection and use of Bio-active™ archwires for individual patient treatments.

## 1. Introduction

Orthodontic archwires constitute an indispensable element of the fixed orthodontic technique, wherein tooth movement is facilitated by the archwire’s forceful interaction with the brackets. The magnitude of the force of this interaction is critical, as excessive force could lead to delayed or irreversible root resorption, while insufficient force may fail to elicit a biological response [1]. Light continuous forces are generally considered more effective in achieving desired tooth movements compared to heavy forces. To generate mild continuous forces, the archwire must possess a wide range and low stiffness. Clinicians can manipulate three factors that influence stiffness: the archwire alloy, the wire’s cross-sectional dimension, and the length of the free wire between brackets [2].

The success of orthodontic therapy using archwires is contingent upon the selection of appropriate archwires and the administration of treatment in phases. Currently, there is no universally perfect archwire for every stage of fixed appliance therapy [3]. The primary objective of the initial archwire, which is placed into the fixed device at the onset of therapy, is to address minor tooth rotations and crowding. Ideal forces are those that are light and continuous, as they are believed to yield predictable and controlled tooth movement while minimizing damage to the teeth and supporting tissues [4,5,6,7]. Clinically, optimal forces facilitate rapid tooth movement while minimizing root resorption [3]. During the era when stainless steel (SS) was the primary alloy used in orthodontics, stiffness reduction for a given application involved either altering the wire’s size or increasing its length through the addition of loops [2]. This traditional wire progression often resulted in underpowered or overpowered teeth due to multiple rounds of increasing size and force. However, the advent of NiTi archwires revolutionized orthodontic treatment in the latter half of the last century [8].

Nickel–titanium (NiTi) wires, initially composed of stabilized martensitic alloy (e.g., original NiTi), evolved over time. Successive generations of NiTi wires included superelastic-active austenitic NiTi alloys, thermodynamic-active martensitic NiTi alloys, and, most recently, graded thermodynamic multi-force NiTi alloys (archwires made from this alloy have three separate areas—frontal, premolar, and molar—of differing hardness, which set them apart from other NiTi archwires) [9].

These advancements are valuable as they allow for the application of physiologically suitable stresses to each tooth, thereby minimizing avascular necrosis and shortening the lag phase [10]. When selecting a multi-force NiTi archwire for treatment, it is essential that we understand how its properties may change over extended clinical use. However, clinicians typically rely on information provided by manufacturers, which often inaccurately describes anticipated force values from the archwire [11].

The literature reviewed in this work deals specifically with the Bio-active™ multi-force archwire, and its examination in an unused state and after use for clinical treatment. The historical chronology of multi-force NiTi archwires was also traced and scientific information was gathered regarding multi-force archwires that are closest to the Bio-active™ one, be it in terms of their properties, composition, or generation.

The present review, without making a claim for full comprehensiveness, concentrates on studies of multi-force NiTi archwires, encompassing mechanical and thermal testing, along with their physicochemical characterization. Its aim is to make the information derived from these studies readily accessible for reference with regard to the selection and use of the Bio-active™ archwire for individual patient treatment.

## 2. Brief Historical Overview of NiTi Archwires

The use of NiTi alloy originated from the space program research conducted by metallurgist W.J. Buehler at the U.S. Naval Ordnance Laboratory in the early 1960s. He is credited with the discovery of the NiTi alloy [12,13]. The first such alloy was developed by Buehler using titanium and nickel in approximately equal atomic proportions (55% Ni: 45% Ti), which he named Nitinol, an acronym for Nickel Titanium Naval Ordnance Laboratory. Buehler observed that the alloy exhibited unique thermodynamic properties when heated, shaped precisely, and, subsequently, cooled. A notable feature not commonly observed in other metals is the alloy’s ability to be easily plastically deformed at low temperatures, and then restored to its original shape simply by raising the temperature [12]. Additionally, Buehler identified several other significant properties of NiTi. Compared to stainless steel (SS), NiTi is approximately 3.5 to 7 times less rigid [14], possesses excellent springback [7], and demonstrates remarkable resistance to irreversible deformation. The superior corrosion resistance and biocompatibility of this alloy render it an excellent choice for intraoral applications [15].

George F. Andreasen introduced NiTi archwires into orthodontic practice in 1976 following Buehler’s discovery of the NiTi alloy [16]. Initially marketed under the name Nitinol, the Unitek Company was the first to commercially introduce NiTi wires. Due to cold working, the original Nitinol wire lacked superelasticity and did not exhibit phase transition characteristics [17]. Despite its absence of shape memory and superelasticity (thus termed “stabilized martensitic” compared to other archwires available at the time), its low stiffness (20% that of SS) and expanded operating range (2.5 times that of SS) contributed to its widespread adoption [18]. Clinicians appreciated its ability to accommodate even the most complex cases without irreversibly distorting. Since then, a wide range of NiTi wires has become available, and the term NiTi now encompasses the entire family of these archwires [15].

**Figure 1:**
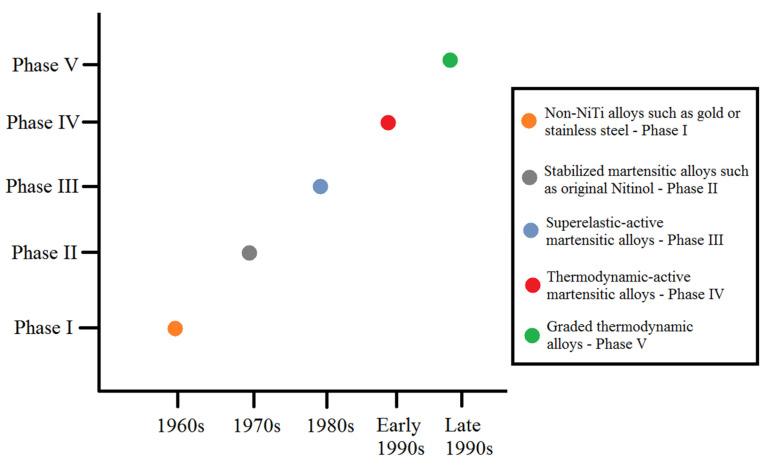
Historical chronological classification of the development of orthodontic archwires.

The chronological classification of orthodontic archwires proposed by Evans and Durning in 1996 relates each phase to a specific mode of force delivery [9]. This classification remains actual to this day (Figure 1).

### 2.1. The First Superelastic Wires

The first superelastic wires were developed in the early 1980s at Furukawa Electric Co. Ltd. in Tokyo, Japan and the General Research Institute for Nonferrous Metals in Beijing [17,19]. These wires, which had similar compositions, eventually became known as Japanese and Chinese NiTi wires, respectively. These superelastic wires are austenitic at typical oral temperatures because their temperature transition range (TTR) is adjusted below oral temperature, but they exhibit stress-induced martensite (SIM) under stress [20]. They provide consistent force levels at increasing deflections (superelastic plateau) and possess exceptional springback qualities (four to five times that of SS) [21,22]. With advancements in manufacturing techniques, it became possible to reduce the TTR and create alloys that, unlike the pseudoelasticity of the previous generation of NiTi, also display thermoelasticity and the heat-induced shape memory effect [9]. The austenite finish (Af) of thermodynamic active martensitic NiTi wires is above room temperature, whereas active-austenitic NiTi wires are completely austenitic at and above room temperature. As a result of the improved TTR control, producers are now able to create wires with the same diameter but varying force levels. Examples of thermodynamic archwires include Sentalloy and NeoSentalloy (NS) from GAC, which are available in heavy-, medium-, and low-force varieties depending on their individually adjusted Af.

### 2.2. Thermodynamic Wires

Thermodynamic wires are also classified as copper NiTi (CuNiTi). They emerged from the discovery that NiTi alloys became more responsive to oral temperatures with the addition of tiny amounts of copper. Three types are similarly available: CuNiTi 27 °C, CuNiTi 35 °C, and CuNiTi 40 °C. These names indicate their Af temperatures and correspond to heavy, medium, and mild force delivery, respectively [9,23]. It is believed that their characteristics offer several advantages over those of earlier generations. Typically, they produce 25–30% of the force that active austenitic archwires do per unit of area [9]. However, thermodynamic wires have drawbacks such as being more costly and highly susceptible to manufacturing procedures. If they are not manufactured appropriately or are used in small diameters, they may be ineffective and deliver little or no force in the unloading curve [24]. Additionally, unlike ordinary non-thermal wires, it is not possible to “feel” the amount of force the wire may be exerting intraorally [2]. It is noteworthy that thermoelastic wire producers usually report force values produced at 35 °C to 37 °C, which are considered reflective of an average person’s mouth temperature [25]. The intraoral temperature of mouth breathers is often lower, between 30 and 33 °C; therefore, thermodynamic wires with a lower Af may be required to provide the necessary force levels. In fact, research by Sakima et al. [24] demonstrated that extremely “light” rectangular thermal NiTi wires, such as Ormco’s CuNiTi40 °C and NS F200g, did not exert any force during a bending test at temperatures below 35 °C, indicating that they might not be suitable for mouth breathers.

### 2.3. Graded Thermodynamic NiTi (GT-NiTi) Archwires

**Figure 2:**
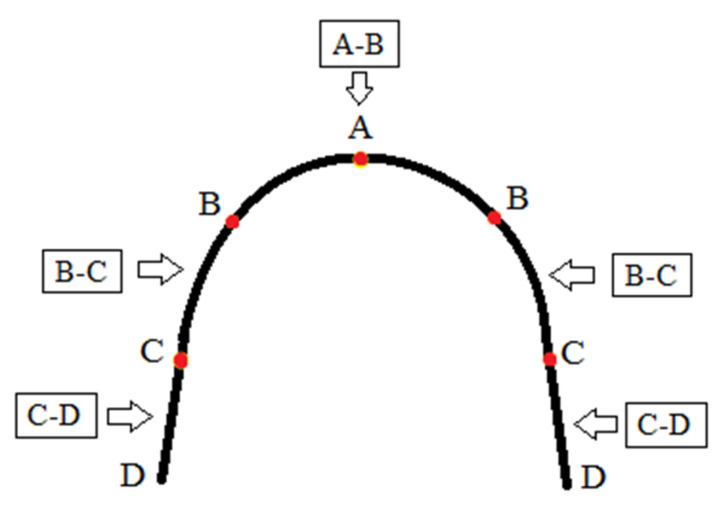
Regions and forces released of a typical multi-force NiTi archwire, where: A–B—frontal region, weakest forces released; B–C—premolar region, medium forces released; C–D—molar region, strongest forces released.

Graded thermodynamic NiTi (GT-NiTi) archwires, also known as multi-force NiTi archwires, were developed based on the concept that the force magnitude should correspond to the tooth’s surface area to achieve consistent stress that maximizes the velocity of tooth movement [2]. Specifically, anterior teeth may be moved more effectively than posterior teeth, requiring significantly lower forces (which is why these archwires are segmented into three regions—frontal, premolar, and molar—as shown in Figure 2). They release biologically tolerable forces along their length, progressively increasing in a front-to-back direction. This concept aims to apply increasingly stronger forces from the anterior to posterior regions of the archwire of uniform dimensions [9]. The development of these archwires became possible with the introduction of direct electric resistance heat treatment (DERHT).

Today, many brands, including “BF” (GAC, Barranquilla, Colombia), “Titanol Triple Force” (Forestadent, Pforzheim, Germany), “Variable Force 3” (Ortho Organizers Inc.—Henry Schein, Carlsbad, CA, USA), “TriTanium” (American Orthodontics, Sheboygan, WI, USA), “Bio-active” (TOMY Inc., Tokyo, Japan), and others, offer their own versions of GT-NiTi archwires.

The “BF” archwires were the first Phase V NiTi archwires introduced to the market [9]. Currently, they are available in square or rectangular cross-sections. According to the manufacturer, a three-point bending test deflected to 2 mm over a 14 mm span at body temperature will yield approximately 80 g of force at the incisor portion, 180 g at the premolar level, and 280 g at the molars for a 0.016 × 0.022″ archwire [26]. It is indicated that the force level increases incrementally from the anterior to the posterior regions.

Forestadent’s “Titanol Triple Force” offers three force segments in an archwire. Very light forces act on the anterior region, medium forces on the canines and premolars, and stronger forces on the molars. This arrangement facilitates the application of a biologically beneficial amount of force to align the teeth. The Titanol Triple Force archwires feature an excellent surface structure with very low friction values [27].

The “Variable Force 3” archwire is a heat-activated, multi-force nickel–titanium archwire with three distinct force regions. The archwire is easy to place because the thermal mechanics allow the wire to remain soft when not in the mouth. Once placed into the warmth of the mouth, the wire takes on its memory shape. This thermal NiTi archwire undergoes a multi-step manufacturing process that consistently creates three different zones of forces along the arch. The Variable Force 3 archwire provides forces ranging from 50 grams in the anterior region to 300 grams in the molar region, depending on the archwire size [28].

Initially, the TriTanium™ (NiTi) alloy was utilized in orthopedics due to its high porosity and favorable biocompatibility [29]. This alloy’s three zones of flexibility in the anterior, mid-region, and posterior parts make it beneficial in orthodontics. An archwire capable of applying forces appropriate for each zone is considered more successful, given the significant differences in the root systems of the teeth in these segments. This is particularly relevant during the initial stage of fixed appliance therapy. Because multi-force orthodontic archwires decrease the incidence of root resorption, it is believed that patient comfort improves [30,31].

The Bio-active™ model multi-force archwire belongs to the latest generation of graded thermodynamic NiTi archwires. Its usage has increasingly expanded in orthodontic practice in recent years, necessitating a better understanding of how clinical use can affect both its mechanical and thermal properties. This understanding enables practicing clinicians to make informed decisions regarding when and how to use it in their patient treatments. Being made of NiTi alloy, the Bio-active™ archwire possesses a high corrosion resistance, biocompatibility, and an inherent ability to osseointegrate [32]. As a dental archwire, it is an essential component of the fixed technique (brackets). Engaging the archwire generates activating forces over an extended period, initiating tooth movement and remodeling. These forces are low and constant [33,34], stemming from the two unique properties of the NiTi alloy: superelasticity and the shape memory effect [35,36]. The Bio-active™ archwire is available in dimensions of 0.016 × 0.016, 0.016 × 0.022, 0.018 × 0.018, and 0.021 × 0.022 inches. This variety allows orthodontists to initiate treatment with a larger-diameter archwire to better control the buccal–lingual position of the front teeth. The archwire’s biomechanical properties (shape memory, force, and superelasticity) can also be attributed to heat-activated archwires. Its shape memory applies progressive forces from the midline to the posterior sections [37,38,39,40].

## 3. Thermal Properties of Multi-Force NiTi Archwires

**Figure 3:**
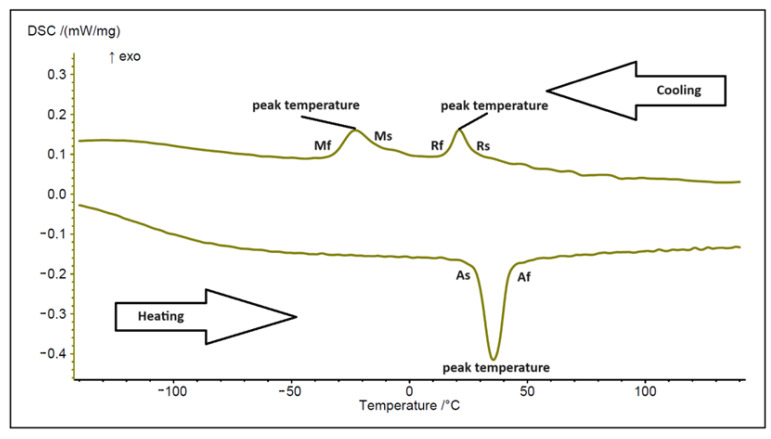
Example of a DSC thermogram of a NiTi orthodontic archwire segment. The starting and finish temperature of each phase transition were determined from lines tangent to the DSC curve, in which there was a deviation of the adjacent baselines [40].

The majority of published orthodontic studies utilize Differential Scanning Calorimetry (DSC) to test the transformation temperatures of orthodontic wires. Additionally, standards for orthodontic wires, specifically ANSI/ADA Standard No. 32—Orthodontic Wires. ANSI/ADA: Chicago, IL, USA, 2017 and ISO 15841:2014, Dentistry—wires for use in Orthodontics. ISO: Geneva, Switzerland, 2014, specify DSC as the method for determining the austenite finish temperature (Af) for orthodontic archwires. The beginning and ending temperatures of the various phases are particularly instructive. Figure 3 displays a DSC thermogram of a sample of a NiTi archwire section in both heating and cooling modes. The impacts of the matching phases are readily discernible. The starting temperature (As) indicates the temperature at which austenite begins to form from martensite when heated. Af is the temperature at which the austenite transformation finishes. The transition temperatures of these phases are starting austenite (As), finish austenite (Af), starting rhombohedral (Rs), finish rhombohedral (Rf), starting martensite (Ms), and finish martensite (Mf), which are determined by specialized software.

Another method that can also be used is the bend and free recovery (BFR) test method, which is a straightforward and useful tool for determining the transition temperatures of heat-activated NiTi orthodontic archwires, according to a study by Obaisi et al. [41]. In general, BFR testing had an average standard deviation that was marginally lower than DSC testing. Additionally, the average discrepancies between the Af temperatures measured by the BFR and DSC test techniques ranged from 0.0 °C at the low end to 2.1 °C at the high end, indicating comparable temperatures. However, the BFR approach is far more cost-effective. In orthodontic treatments, the phase transitions from martensite to austenite and vice versa can be advantageous if the TTR for NiTi alloys is narrow enough. Changes in the ratio of nickel to titanium or the addition of minor amounts of additional elements [31] can reduce this range. Therefore, the characteristics of the orthodontic archwires are influenced by their elemental composition [38]. Because DSC enables the determination of the phase transition temperature and the amount of energy emitted or absorbed during the heating or cooling processes, it is one of the most helpful techniques for assessing the TTR of orthodontic archwires [42,43]. These investigations focus on the relationship between temperature variations and the thermomechanical characteristics of NiTi alloy, particularly the phase changeover temperatures [17,44,45,46,47,48]. In addition to influencing the mechanical properties, specific heat treatment in the designated areas of the archwire results in a change in the modulus of elasticity and the distinct transition temperature range (TTR) in the three locations (the lowest in the frontal area, increasing at the premolar, and the maximum at the molar). Without altering the size of the cross-section, this causes a varied force release throughout its length [19]. Superelasticity rather than the “shape memory effect” was noted for NiTi archwires with austenite finish (Af) temperatures set significantly below the oral temperature, as no thermal transition between the two phases took place [49]. The Af can be adjusted to be nearly at room temperature [50] or intraoral temperature by employing a certain manufacturing method.

## 4. Mechanical Properties of Multi-Force NiTi Archwires

The type of alloy has been crucial in selecting an archwire since their introduction [18]. The mechanical characteristics of any given NiTi wire are primarily determined by the manufacturer’s processing and parameter settings [24,51]. Stress-induced martensite (SIM) occurs in NiTi thermoelastic alloys when stress is applied to the parent phase material. The distorted substance returns to the parent phase and its original shape upon unloading. This stress does not change much over a wide range of wire activation. Due to the mechanical characteristics of superelastic NiTi, continuous stresses can be applied to the teeth throughout extended activation periods, eliciting a desired biological response [21,52,53]. For example, Lombardo et al. [54] published a study in 2018 which showed that multi-force NiTi archwires displayed 27% and 31% lighter mean forces in the upper and lower arches, respectively, in addition to 62% and 40% reductions in the unloading plateau slope and length, respectively, compared to conventional CuNiTi wires.

Two of the more widely used methods to test the mechanical properties of NiTi archwires are nanoindentation and three-point bending. The nanoindentation test has the advantage of providing both elastic modulus and hardness data from a single test; however, it requires that the surface of the studied sample be completely flat [55]. In one of our previous studies [38], we used dynamic nanoindentation to test the indentation hardness and modulus of the Bio-active archwire. The test had to be modified, as the sample had a high surface roughness, causing problems for the indenter to detect the surface. The results showed that, as the time of clinical use increased, the indentation moduli and hardness decreased. We also conducted a similar study [56] on the TriTanium archwire, which revealed significant differences in the mechanical properties among all regions of the archwire. The indentation hardness and indentation modulus of the posterior region were found to be the highest, while those of the anterior region were the lowest. Additionally, similar to the study of the Bio-active archwire, an inverse proportionality was observed between the time of clinical use and the indentation hardness and modulus.

The main advantage of a three-point flexural test is the ease of specimen preparation and testing; however, the results are sensitive to the specimen and loading geometry, as well as strain rate, and it does not measure fundamental material properties [57]. Ibe and Segner [58] were the first to use a three-point bending test to measure the force release of the archwires they studied. They found that Forestadent’s Titanol Multiforce archwire releases greater force in its molar region compared to the premolar or frontal regions.

**Figure 4:**
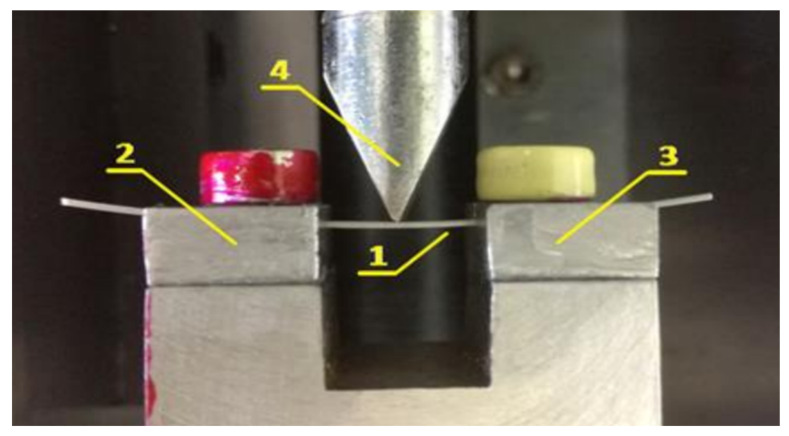
Modified three-point bending test apparatus. Position 1 indicates the sample being tested; Positions 2 and 3 indicate the specially made incisions in the props; and Position 4 indicates the indenter [39].

Subsequently, other authors, such as Sanders et al. [59], have also employed a three-point bending test to examine NiTi archwires. We have also conducted a study [39] on the mechanical properties of the Bio-active and TriTanium archwires using a specially modified three-point bending apparatus fixed to a standard LMT-100 (LAM Technologies, Firenze, Italy) for physical–mechanical testing (Figure 4). The slot design limits the possibility of undesired deformation processes, ensuring the concept of “controlled” free contact between the orthodontic archwire and the supports without entirely eliminating the chance of slipping during loading.

The available literature on commercially studied NiTi wires is not exhaustive. It largely pertains only to unused archwires, despite several independent studies [24,51,58,60,61,62,63,64,65,66] attempting to address this issue by comparing the mechanical properties of various NiTi archwires in vitro under the same conditions.

## 5. Overview of Various Studies on the Bio-Active™ and Contemporary Multi-Force NiTi Archwires

### 5.1. Bio-Active™ and TriTanium™ Multi-Force NiTi Archwire: Physicochemical, Mechanical, and Thermal Properties

**Figure 5:**
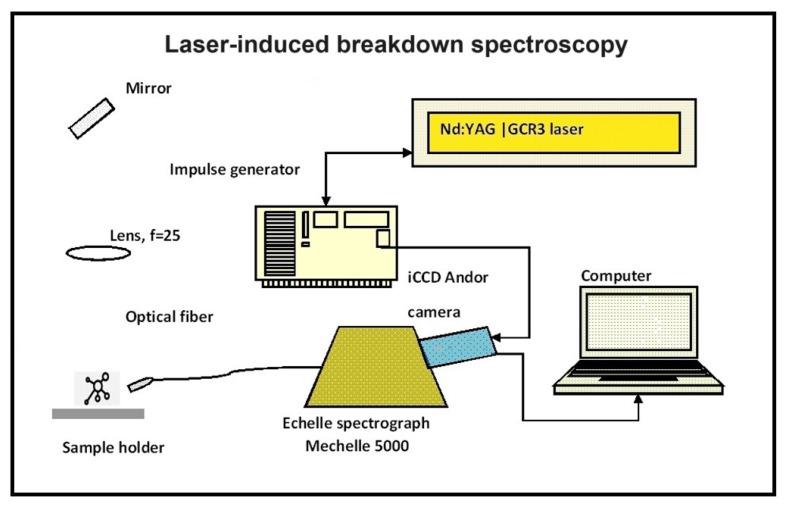
Scheme of LIBS apparatus; adapted from Rodrigues et al., 2020 [72].

As mentioned, the NiTi multi-force Bio-active™ archwire releases biologically tolerable forces along its length in a front-to-back direction, which increase along it. The frontal segment releases the weakest force, the premolar segment releases greater force, and the molar segment has the greatest force release. The manufacturer of the Bio-active archwires provides a characteristic of the wire’s expected properties [67]. It should be noted that this characteristic only applies to the wire in an unused state and does not account for potential changes during clinical use. It is important that independent studies be conducted to verify the information provided by the manufacturer, as manufacturers generally do not openly discuss their proprietary manufacturing techniques. To address this, we carried out studies [38,39,40,68,69], where the mechanical and thermal properties of the Bio-active, along with the TriTanium archwires, were studied and compared. Part of these studies also includes physicochemical characterization [38], which involves tests such as X-ray diffraction (XRD), energy-dispersive X-ray spectroscopy (EDS), scanning electron microscopy (SEM), and laser-induced breakdown spectroscopy (LIBS). The LIBS test (Figure 5) specifically has a wide area of application due to its ability to perform a microprobe elemental analysis of a wide variety of samples with no preliminary sample preparation. LIBS allows for an elemental analysis of a wide range of samples without preparation. The method is implemented by focusing a powerful laser beam on the sample through a lens of 25 cm focal length. The laser source is a pulsed nanosecond laser—Nd:YAG Quanta Ray GCR3, generating a wavelength of 1064 nm, a repetition frequency of 1 Hz, and a pulse energy of 30 mJ. The focused laser beam ablates the sample, yielding laser-induced plasma. The light emitted by the plasma is transmitted through the optical fiber to the registration system—Mechelle spectrograph 5000 equipped with Andor iStar ICCD camera. The system uses special software—Andor SOLIS 4.13. By analyzing the spectrum of the plasma and registering specific spectral lines for each element, it is possible to obtain information about the elements in the target sample.

It shows that the Bio-active archwire contains trace amounts (0.3 wt%) of Fe and Cr [38,68], while the TriTanium archwires do not contain any elements other than Ti and Ni [68]. The presence of Fe and Cr should be taken into account when treating patients allergic to those elements.

Similar microelement findings are reported in a study [70] that investigates round and rectangular thermally activated CuNiTi dental archwires, before and after clinical use up to 6 weeks and past 8 weeks. CuNiTi wires come in three temperature options: 27 °C, 40 °C, and 35 °C, corresponding to the upper austenitic temperatures that finalize the transformation from martensite to austenite [17]. According to EDX’s element analysis, the three thermally activated archwire variants are similar in that they contain roughly 44 wt% nickel, 51 wt% titanium, small amounts of copper (5 wt%), and 0.2 to 0.3 wt% chromium in place of titanium [71]. According to Kusy [8], these arches have a minimum of 5–6 wt% copper and 0.2–0.5 wt% chromium. In order to counteract the effect of copper on the rise in transition temperature in the mouth, the 40 °C form of the archwire has 0.2 wt% chromium, whereas the 27 °C variant has 0.5 wt% chrome. The results of the tests show that, both after the treatment period of up to six weeks and after eight weeks, there are no appreciable changes in the chemical composition of the surface of the thermally activated wires (both round and rectangular cross-section). The timeframe includes the average amount of time that the patient’s orthodontic wires are in their mouth. Our investigations of the Bio-active™ archwire’s chemical composition show similar results—the Ni and Ti wt% remain unchanged even after prolonged use, and trace amounts of Fe and Cr were registered in all regions of the archwire [38].

The deactivation force, also known as the unloading force, of the appliance is what acts to level and align the teeth, not the activation force. Hence, knowledge of the deactivation behavior is important to the clinician for optimal wire selection. In our research, we have focused on the role of deactivating forces in the unloading phase, and we have found that the results obtained in the archwires’ activation phase are logically higher than in the deactivation phase [39]. Throughout the course of use, no statistically significant variations in the Af are observed in the various segments of the Bio-Active and TriTanium^®^ archwires. All tested groups (not used and used for up to and over 8 weeks) have the same Af [40]. We found that the Bio-active™ wires preserve their properties, both mechanical and thermal, over an extended period of clinical use (over 8 weeks), with no statistical differences found between all tested groups (unused and used up to and over 8 weeks) [39,40]. They can be classified into the group of martensite-active wires (heat-activated) because the manufacturers set the Af to be above room temperature and close to the temperature of the oral cavity. In contrast, the TriTanium archwires show different thermal transition temperatures for the austenitic, martensitic, and R phases between the as-received and clinically retrieved TriTanium archwires [68]. As their clinical use time increases, an Af temperature difference in all regions of the archwire, especially in the premolar region, can be seen at ~8 °C. Their Af temperature is also set quite below the temperature of the oral cavity. This means that, at room temperature, before engaging the archwires into the bracket slot, they are already in the austenitic phase [40]. A summary of recommendations for the clinical use of the Bio-active™ and TriTanium™ archwires based on the reviewed literature is presented in Table 1.

### 5.2. Multi-Force NiTi Archwires

Another study, in which the Bio-active archwire is examined alongside others, was conducted by Sanders et al. [59]. In this study, six wires were examined: four multizone archwires—DuoForce (Forestadent, Pforzheim, Germany), TriTanium (American Orthodontics, Sheboygan, WI, USA), Triple Force (ODS, Kisdorf, Germany), Bio-Active (GC, Breckerfeld, Germany), and two multistrand products without force zones—a nine-strand NiTi, TurboWire (Ormco, Orange, CA, USA), and an eight-strand stainless steel (SS) wire, Multibraid (GAC, Dentsply Sirona, York, PA, USA), using a three-point bending test. The results indicate that the maximum median forces of unused TriTanium (5.3 N), Triple Force (4.6 N), Bio-Active (5.0 N), and DuoForce (5.1 N) are higher than the forces of the NiTi TurboWire and the eight-strand Multibraid. On the other hand, the tests confirm the existence of multiple force zones in the NiTi wires for forces and torque-moments, respectively. Since the torque-moments of the multizone wires are rather high, it is not recommended to use these wires as a first “leveling wire” in orthodontic treatment, especially in extremely crowded cases.

An examination and evaluation of the temperature transition range (TTR) of seven types of commercially available 0.019″ × 0.025″ thermally activated nickel–titanium archwires, Nitinol Termoativado (Aditek, Cravinhos, SP, Brazil); NeoSentalloy F200 (GAC, Bohemia, NY, USA); Thermo Plus (Morelli, Sorocaba, SP, Brazil); Copper NiTi 35 °C (Ormco, Glendora, CA, USA); Flexy Thermal 35 °C (Orthometric, Marília, SP, Brazil); Superthermal Nickel Titanium Arches (Orthosource, Matão, SP, Brazil), and Heat Activated NiTi (Highland Metals, San Jose, CA, USA), was conducted by Spini et al. [42]. It was found that all thermally activated NiTi archwires analyzed presented a stage transformation during thermal scanning, with a final austenitic temperature (Af) ranging from 20.39 °C to 45.42 °C. Three of the brands (Nitinol Termoativado, Thermo Plus, and Superthermal Nickel Titanium Arches) presented an Af close to room temperature and, as such, do not possess shape memory and pseudoelasticity, both of which are desirable in clinical applications. The thermally activated NiTi archwires show great variability in the TTR, and the elastic parameters of each NiTi archwire should be provided by the manufacturers to allow the achievement of the best clinical performance possible.

The BioForce GAC archwire has an Af near the intraoral temperature in its frontal part, as shown by Rodrigues et al. [72]. This indicates that this section can be easily placed into teeth with high rotations because it has a shape memory effect and is more elastic at ambient temperature. The archwire hardens, changes into austenite, and regains its shape when the temperature rises. Roulias et al. [73] also studied the mechanical and thermal characteristics of the front and posterior segments of this archwire and found that the Af temperature is higher than the average intraoral temperature and that the forces released exceed the medically permissible limits, even at 2 mm deflections. Both studies were conducted on unused archwires and are important for their clinical use.

Two types of unused archwires, Variable Force 3 (Ortho Organizers Inc.—Henry Schein) and TriTanium (American Orthodontics), had their mechanical properties compared by Cherneva and Petrunov [67] using a nanoindentation test. The study shows that, despite the identical data provided by their manufacturers about these two archwires, there are differences in their properties. Specifically, the TriTanium archwire exhibits a higher indentation hardness and modulus compared to the Variable Force 3™ archwire consistently. This difference can be attributed to the higher concentration of titanium in the elemental composition of the TriTanium archwire. The multi-step manufacturing process, which consistently creates three different zones of forces along the archwire, can account for the observed differences in relation to the regions of the archwires.

Also of interest is a study by Friedli et al. [74], which investigates how storage temperature influences the mechanical properties of NiTi, CuNiTi, and stainless steel (SS) orthodontic wires. Three variations of CuNiTi (Cu-NiTi 27 °C, 35 °C, and 40 °C), along with stainless steel (SS) and nickel–titanium (NiTi), all with a size of 0.017 × 0.025 inches, are examined using a three-point bending test in a chamber that had been preheated to 36 °C. The orthodontic wires are kept at four distinct temperatures (5 °C, 22 °C, 36 °C, and 60 °C) for twenty-four hours prior to the mechanical testing. The obtained results demonstrate that, after being stored at various temperatures, the mechanical forces exerted by CuNiTi 27 °C wires exhibit the most stable behavior, whereas CuNiTi 35 °C wires show the most fluctuation. Stainless steel exhibits no changes in its mechanical characteristics when bent, as would be expected. This study highlights the importance of regulating the storage temperature of orthodontic archwires to avoid unexpected changes.

In addition to that, in-depth studies are conducted to this day which aim to establish a structure–property–performance correlation in contemporary biomedical archwires via a comprehensive characterization of the physical–chemical–mechanical properties and to relate to the thermo-mechanical history. These works focus mostly on the two most widely-used biomedical Ti-containing alloys: NiTi and β-Ti. The novel methodology described is evidenced as generating a predictive profile of the eventual biomechanical properties and practical performance of the commercial materials. One such work encompasses a reproducible and comprehensive approach expected to aid in the future optimization and rational design of devices of metallic origin [75].

In 2023, Alcaraz et al. [76] studied five different NiTi archwires from five different manufacturers (GAC, 3M, ODS, GC, and FOR) using a three-point bending test with the aim of describing and statistically determining the changes to their superelastic properties following clinical use and sterilization. The study found that, following three months of clinical use, FOR exhibited a longer activation period and a larger release of force. The mechanical characteristics of the GC, EURO, and FOR archwires appear to be lost. After being used in a clinical setting, the GC wires released a greater force than other brands. The GAC, 3M, and FOR wires regained their superelasticity following disinfection; however, the EURO archwires lost more. The GAC, 3M, and FOR wires regained their characteristics upon disinfection for smaller deformations (<2 mm); however, the EURO archwires seem to lose their superelasticity. Significant differences can be seen in the superelastic characteristics and released forces across all investigated groups.

An important issue addressed by a study [77] in 2024 is the lack of standardization of the subjects and force. It seeks to remedy this by conducting a standardized in vivo quantitative assessment of the working range of different orthodontic archwires and their effect on root resorption, which, it claims, has not been previously attempted. The study quantitatively compares and assesses these properties in a standardized split-mouth design using a sample of 10 Wistar rats. CuNiTi wire is used to apply a 25 g force to one of their two upper incisors, whereas a NiTi wire is used to apply the same force to the control side. Cone-beam computed tomography is utilized before and after application to take images for comparing working range and root resorption. Taking constant forces into account, it is demonstrated that CuNiTi wires have a greater operating range than NiTi because of the material’s more horizontal force-deflection curve, providing a continuous, steady, and almost constant force along the tooth’s transition path during movement. Consequently, CuNiTi wires are deemed superior to NiTi wires during orthodontic therapy, as long as the producer complies with ADA/ANSI specification number 32 for orthodontic wires. This allows CuNiTi wires to remain in the patient’s mouth for a longer period with fewer replacements needed, potentially reducing the number of appointments and the overall cost of therapy.

### 5.3. General Characteristics of the Studies

All studies included in this section reflect the physicochemical, mechanical, and thermal properties of multi-force NiTi archwires, with an accent on the Bio-active™ archwire. The mentioned studies investigate both as received archwires and archwires after clinical use. Table 2 presents a summary of these studies.

An interesting approach to patient treatment, which includes archwires, is proposed in a study [78], which proposes the use of appropriate dental CAD software (V24.0, 2020). It notes that the biggest advantage this gives is appliances designed in that way and printed from metal can have their support points planned on places that are close to the level or in the area of the centre of resistance of the tooth or group of teeth that are supposed to be moved. This allows for movement that is close to the corps and keeps the support zone unchanged. Another study [79] explores the use of artificial intelligence (AI) in dental practice and how patients feel about it. AI can be effectively used to process large volumes of data, thus reducing the workload of the practicing orthodontist significantly, especially when it comes to the potential use of dental CAD software as mentioned previously. There are, however, some issues, mainly relating to the AI systems inheriting biases from the datasets used to train them and privacy issues. The study focuses on patients in Bulgaria, finding that people there still rely more on human decisions, experience, and knowledge. Due to these attitudes, the authors conclude that digitalization should be done gradually, so that patients can perceive the change step by step and be able to actively participate in it.

From the current review, it can be observed that studies on the mechanical, thermal, and physicochemical properties of both unused and clinically used multi-force archwires, including the Bio-active archwire, contribute additional information beyond that provided by the manufacturers, enriching the knowledge regarding them. We also noticed that very few of the studies that we encountered take note of the patient age groups, their lifestyle, and other factors. This shows that there is a gap in the knowledge about how those factors influence the archwires and how that may reflect on the treatment of patients. This underlines the necessity of more future studies in this line of investigation.

## 6. Concluding Remarks

Considering that each patient has their own individual needs when it comes to orthodontic treatment, our review, relating to multi-force Bio-active archwires and various commonly used contemporary NiTi multi-force archwires, will help orthodontists make informed decisions regarding which archwire to use for each corresponding patient. This review highlights the following points:

- Multi-force archwires are made of NiTi alloys and have no additional elements mixed in, whereas the Bio-active ones contain traces of Fe and Cr, which should be taken into account for patients with allergies.
- With their graduated biologically tolerable forces, multi-force archwires are particularly suitable for patients with periodontal problems and minimal crowding.
- In general, multi-force NiTi archwires release progressively increasing forces in a front-to-back direction along their length, while CuNiTi wires have a greater operating range than NiTi due to the material’s more horizontal force-deflection curve, providing a continuous, steady, and almost constant force along the tooth’s transition path during movement.
- Over a period of up to and exceeding 8 weeks, multi-force archwires (Bio-active and TriTanium) maintain their mechanical properties, reducing the need for patients to visit their treating orthodontist each month.
- The thermal properties of multi-force archwires depend on external factors, allowing patients to regulate the released forces by consuming cold or warm foods and drinks. Additional cooling can enable the use of Bio-Active and TriTanium archwires as, first, leveling archwires, but they are not recommended for patients with mouth breathing.

## Figures and Tables

**Table 1:** Summary of recommendations for the clinical use of the Bio-active™ and TriTanium™ archwires.

Bio-Active™		TriTanium™	
Indicated	Counter Indicated	Indicated	Counter Indicated
Patients do not complain from pain when the archwire is first placed Leveling of teeth arches for small crowding up to 2 mm Patients with periodontal problems, due to the small forces exerted by the archwire Early expression of torque during the first phase of treatment Introducing retained canine teeth in the tooth arch, due to the constant force exerted by the archwire Appropriate for extended clinical use over 8 weeks	Application on mouth-breathing patients prior to removal of the habit Application on mouth-breathing patients with crowding in the front, since no temperature transition between phases can occur Ingestion of cold drinks during the first week of treatment	Leveling of teeth arches for small crowding up to 2 mm Patients with periodontal problems, due to the small forces exerted by the archwire Recommended for mouth-breathing patients Early expression of torque during the first phase of treatment Introducing retained canine teeth in the tooth arch, due to the constant force exerted by the archwire Appropriate for extended clinical use	Ingestion of cold food and drinks during the first week of treatment

For both types of archwires, spray cooling is recommended before use to ensure the archwire can be inserted into the bracket slots.

**Table 2:** Overview of various studies on the Bio-active™ and contemporary multi-force NiTi archwires.

Archwires Studied	Type of Performed Characterization	Clinical Use of Investigated Arcfhwires	Authors and Year Published
Bio-active™ (TOMY Inc., Tokyo, Japan)	Physicochemical and mechanical	As received and clinically used	Gerogieva et.al. (2021) [38]
Bio-active™ (TOMY Inc., Tokyo, Japan) TriTanium™ (American Orthodontics, Sheboygan, WI, USA)	Mechanical	As received and clinically used	Stoyanova-Ivanova et.al. (2023) [40]
Bio-active™ (TOMY Inc., Tokyo, Japan) TriTanium™ (American Orthodontics, Sheboygan, WI, USA)	Thermal	As received and clinically used	Stoyanova-Ivanova et.al. (2023) [40]
DuoForce™ (Forestadent, Pforzheim, Germany) TriTanium™ (American Orthodontics, Sheboygan, WI, USA) Triple Force™ (ODS, Kisdorf, Germany) Bio-active™ (GC, Breckerfeld, Germany) TurboWire™ (Ormco, Orange, CA, USA) Multibraid™ (GAC, Dentsply Sirona, York, PA, USA)	Mechanical	Clinically used	Sanders et.al. (2021) [59]
35 °C Thermo-Active Copper NiTi	Physicochemical and thermal	As received and clinically used	Stoyanova-Ivanova et.al. (2021) [68]
Bio-active™ (TOMY Inc., Tokyo, Japan)	Physicochemical, mechanical, and thermal	As received	Stoyanova-Ivanova et.al. (2024) [69]
Round thermal activated copper–nickel–titanium Rectangular thermal activated copper–nickel–titanium	Physicochemical	As received and clinically used	Petrov et.al. (2015) [70]
35 °C Thermo-Active Copper NiTi	Thermal	As received	Brantley et.al. (2008) [71]
Bioforce™ (GAC, Dentsply Sirona, York, PA, USA)	Thermal	As received	Rodrigues et.al. (2020) [72]
Bioforce™ (GAC, Dentsply Sirona, York, PA, USA)	Physicochemical, mechanical, and thermal	Clinically used	Roulias et.al. (2023) [73]
Ormco stainless steel (SS) Ormco nickel titanium (NiTi) Ormco 27 °C, 35 °C and 40 °C Copper NiTi (CuNiti)	Mechanical	As received	Friedli et.al. (2020) [74]
GC Orthodontics Europe GmbH (Breckerfeld, Germany) Nickel Titanium archwire Nitinol^®^ SuperElastic, Euro NiTi Opto TH Plus Titanol^®^ Superelastic, Sentalloy^®^ superelastic	Mechanical	As received and clinically used	Alcaraz et.al. (2023) [76]
M5™ Thermal Copper NiTi, G4™ Nickel Titanium	Cone beam computed tomographies (CBCTs)	Clinically used	Adly et.al. (2024) [77]

## Data Availability

The data are contained within the article.
